# Modifiable risk factors linked to the development of rheumatoid arthritis: evidence, immunological mechanisms and prevention

**DOI:** 10.3389/fimmu.2023.1221125

**Published:** 2023-09-12

**Authors:** Jeba Atkia Maisha, Hani S. El-Gabalawy, Liam J. O’Neil

**Affiliations:** Manitoba Centre for Proteomics and Systems Biology, Department of Internal Medicine, University of Manitoba, Winnipeg, MB, Canada

**Keywords:** rheumatoid arthritis, prevention, environment, risk factors, smoking, diet, mucosal

## Abstract

Rheumatoid Arthritis (RA) is a common autoimmune disease that targets the synovial joints leading to arthritis. Although the etiology of RA remains largely unknown, it is clear that numerous modifiable risk factors confer increased risk to developing RA. Of these risk factors, cigarette smoking, nutrition, obesity, occupational exposures and periodontal disease all incrementally increase RA risk. However, the precise immunological mechanisms by which these risk factors lead to RA are not well understood. Basic and translational studies have provided key insights into the relationship between inflammation, antibody production and the influence in other key cellular events such as T cell polarization in RA risk. Improving our general understanding of the mechanisms which lead to RA will help identify targets for prevention trials, which are underway in at-risk populations. Herein, we review the modifiable risk factors that are linked to RA development and describe immune mechanisms that may be involved. We highlight the few studies that have sought to understand if modification of these risk factors reduces RA risk. Finally, we speculate that modification of risk factors may be an appealing avenue for prevention for some at-risk individuals, specifically those who prefer lifestyle interventions due to safety and economic reasons.

## Introduction

Rheumatoid Arthritis (RA) is a common autoimmune disease that primarily targets the synovial joints. At the onset of RA, patients present to clinical care with painful and swollen joints, with symptoms typically in the hands and feet (1). Untreated RA leads to the development of bone erosions, joint damage and functional disability. Furthermore, RA leads to considerable systemic inflammation which is associated with premature cardiovascular disease and early mortality. Fortunately, modern advances in therapeutics, specifically the development of biologic medications, has improved outcomes for many patients with RA. In spite of this, RA is considered a life-long disease without a cure, and although medications are effective, they are costly and have the potential to cause side effects. Further, many patients do not respond to RA therapy, leaving them with chronic pain and poor quality of life (2). Although our understanding of the pathogenesis of RA has improved dramatically over the last number of years, the precise etiology of RA remains elusive.

## RA risk factors and progression from a pre-clinical state

It is well understood that the progression into RA occurs in multiple stages, typically over many years. The key genetic risk factor, the shared epitope (SE), is an HLA-DRB1 genetic risk locus (3, 4) that is strongly associated with the development of RA, specifically anti-citrullinated protein antibody (ACPA) positive RA. Studies suggest that the SE binds to citrullinated peptides more efficiently than non-SE HLA-DRB1 variants (5). Other genetic risk single nucleotide polymorphisms (SNPs) have been described, such as *PTPN22* and *PADI4*, pointing towards the involvement of adaptive immune cell signalling (6) and pathogenic citrullination (7) as key steps towards RA development. By in large, the risk imparted by the SE explains the majority (~ 60%) of the genetic risk for RA. RA is also a sex-biased disease, with females being affected more than males at a ratio of 3:1 (8). Being a first-degree relative (FDR) of an RA patient also increases the risk of RA, likely due to both shared genetics and common environmental exposures amongst family members (9, 10).

Ultimately, the combination of these risk factors leads to the development of autoimmunity, marked by autoantibodies which are typically directed towards modified proteins, with citrulline being the best-characterized (3, 9–12). Antigenic targets also include other post-translational modifications such as homocitrulline, the by-product of a process called carbamylation (13), acetylated proteins (14) and malondialdehyde (MDA)/MDA-acetaldehyde (MAA) adducts (15). Rheumatoid Factor (RF), antibodies directed against the Fc portion of IgG, may also be detected at this time. Over time, often years, the immune response matures, with increasing levels of detectable ACPA and accumulation of antibody reactivity to a broader set of proteins (epitope spreading). ACPA has also been shown to undergo variable domain (Fab) N-linked glycosylation, which is closely linked to somatic hypermutation, a process by which the specificity of immunoglobulin is enhanced by T-cell driven maturation of B-cells. Importantly, the glycosylation of ACPA occurs prior to the development of RA, and is seemingly a strong predictor for imminent onset of arthritis (16, 17). Once the ACPA response matures, RA is considered imminent (3), however it is not known what event(s) ultimately triggers the development of inflammatory arthritis from a state of autoimmunity (18).

Aside from non-modifiable risk factors for RA development, much attention has shifted towards modifiable risk factors, specifically lifestyle changes that may reduce the risk of incident RA. This is of specific importance to ACPA+ at-risk individuals where the risk of developing RA may be as high as 45% (10). Herein, we review the modifiable risk factors that are associated with the development of RA and postulate the immunological mechanisms by which this risk is imparted. The focus of the review is on risk factors that are potentially associated with RA risk, namely inhaled exposures, diet and the microbiome. Finally, we review evidence linking modification of these factors with a *reduction* in RA risk, setting the stage for future RA prevention trials in high-risk populations.

## Inhaled exposures

### Cigarette smoking

Cigarette smoking, a widely prevalent habit across the world, has been extensively studied for its impact on human health, specifically respiratory and cardiovascular diseases (19). Of all modifiable risk factors, cigarette smoking is the strongest and best studied contributor of RA risk (20, 21). Multiple case-control studies (22–24) and retrospective cohort studies (25–27) have linked cigarette smoking, both duration and intensity (28) (typically measured by pack-years) with enhanced RA risk (**Table 1**). For example, a meta-analysis of 10 studies showed that in heavy smokers, the risk of developing RA was 2-fold higher compared to never smokers, with evidence of a dose-response based on pack-year exposure. The risk with smoking appears to be much stronger for the development of seropositive RA, rather than seronegative (26, 29), including both RF and ACPA positive RA (30, 36–38). Interestingly, there is an important gene-environment interaction between smoking and the shared epitope risk alleles (21, 30, 39). For example, in the EIRA cohort in Sweden, the odds ratio (OR) of developing seropositive RA in smokers with the SE was substantially higher (SE and Smoking OR 10.0) than the odds for individuals with only 1 (SE alone OR 4.8, Smoking alone OR 1.9) of the 2 risk factors (36). Smoking may also interact with other RA risk allele’s including *PADI4*, which also displays a sex bias toward enhancing RA risk in men (33). Importantly, smoking enhances the risk of developing RA in seropositive at-risk prospective cohorts (34, 35), although this association is somewhat controversial as it has not been replicated in other prospective cohorts (3, 9, 40), possibly due to limited sample size. Interestingly, passive smoking through parental exposure has been shown to be independently associated with RA risk (41), perhaps suggesting that RA risk may never normalize even with complete cessation. Smoking cessation in established RA is associated with reduced disease activity and cardiovascular disease risk (42), further strengthening the association between smoking and RA pathogenesis.

**Table 1:** Selected studies showing an association between cigarette smoking and Rheumatoid Arthritis (RA) risk.

Exposure	First Author	Study type	Cohort	Outcome	Risk of exposure	# RA cases	Reference
Cigarette Smoking	Di Giuseppe	Meta-analysis (3 prospective cohorts, 7 case-control)	Multiple	RF +/- RA	Ever smoking: RF+ RA RR 2.47, RF- RA RR 1.58	4552	(28)
Cigarette Smoking	Hedstrom	Case-Control	Sweden (EIRA)	ACPA +/- RA	Ever smoking: ACPA+ RA OR 1.9, ACPA- RA OR 1.3	3655	(29)
Cigarette Smoking	Too	Case-Control	Malaysia (MyEIRA)	ACPA +/- RA	Ever smoking: ACPA+ RA OR 4.1	1076	(30)
Cigarette Smoking	Bang	Case-Control	Korea	RA	Ever smoking: RA OR 2.7	1482	(31)
Cigarette Smoking	Pederson	Case-Control	Denmark	ACPA +/- RA	Ever smoking: ACPA+ RA OR 1.22 to 57.4; stratified by pack-year exposure AND SE+/-	515	(32)
Cigarette Smoking	Kochi	Case-Control	Japan	RA	Ever smoking: RA OR 1.15 to 1.35	2015	(33)
Cigarette Smoking	Karlson	Retrospective Cohort	USA (Women’s Health Cohort)	RA	Ever smoking: RA RR 1.10 to 1.32; stratified by pack-year	7697	(25)
Cigarette Smoking	Costenbader	Retrospective Cohort	USA (Nurses Health Study)	RA	Ever smoking: RA RR 1.47	680	(26)
Cigarette Smoking	Di Giuseppe	Retrospective Cohort	Sweden (Swedish Mammography Cohort)	RA	Ever smoking: RA RR 2.31	219	(27)
Cigarette Smoking	de Hair	Prospective Cohort	Netherlands (IgM RF or ACPA + at-risk)	RA	Ever smoking: Sero+ RA HR 9.6	15	(34)
Cigarette Smoking	Ponchel	Prospective Cohort	United Kingdom (Leeds; RF or ACPA+ with joint pain)	RA	Ever smoking: Sero+ RA OR 3.1	125	(35)

ACPA, anti-citrullinated protein antibodies; RF, Rheumatoid Factor; EIRA, Epidemiological Investigation of Rheumatoid Arthritis; RR, Relative Risk; OR, Odds Ratio; HR, Hazards Ratio. All RR/OR/HR are statistically significant unless otherwise stated.

### Cigarette smoke influences RA autoantibody development

The mechanisms by which smoking influences RA development remain unclear. Smoking is strongly associated with the development of RA-specific autoantibodies in unaffected individuals, suggesting a role in breaching immune tolerance. Interestingly, the association between smoking and seropositivity suggests that there may be an influence on the development of both RF and ACPA (43). With respects to RF development, mice exposed to chronic cigarette smoke preferentially develop RF, rather than ACPA. Furthermore, humans with cigarette smoke related lung disease develop RF, as opposed to ACPA (44). In patients with RA, smoking is associated with RF seropositivity, specifically IgA and IgM isotypes (45). In the EIRA cohort, the strength of association between smoking and RA was highest for ACPA+/RF+ (OR 2.0), followed by ACPA-/RF+ (OR 1.6). A positive but nonsignificant association with RF-/ACPA+ RA was observed (OR 1.2), again pointing towards the importance of understanding the relationship between RF, ACPA and smoke exposure (36).

In asymptomatic FDR (First-Degree Relatives) of RA patients and non-RA individuals with joint pain, the association between smoking and ACPA is actually stronger than the association between HLA-SE and ACPA (46). Interestingly, in individuals who are seropositive with joint pain, the effect of smoking on inflammatory arthritis onset is less clear, with HLA-SE potentially playing a more dominant role at this stage (46). Smoking may influence the development of autoantibodies at the mucosal surface (47) through the enhanced production of Peptidyl Arginine Deiminase (PAD) (48), the human enzyme responsible for citrullinating proteins, including known RA autoantigens such as vimentin (49). Enhanced citrullination in the lungs (21, 48) may lead to aberrant autoantibody responses and the development of pulmonary ACPA+ B cell (50). Spontaneous NET formation, a potential source of PAD, is detectable in lungs of individuals at-risk to develop RA which is also associated with sputum IgA ACPA (51) (mucosal ACPA). NETs themselves are decorated with citrullinated proteins (52) and may be responsible for autoreactivity to citrullinated histones in RA patients (53). Some mouse models of RA have corroborated the effects of cigarette smoke exposure on inflammatory arthritis (54) and ACPA development (55). It should be noted however cigarette smoke has been shown to reduce CIA arthritis in some studies (56), a finding that is likely due to the timing of smoke exposure (57).

### The effects of cigarette smoking on innate and adaptive immunity

**Figure 1:**
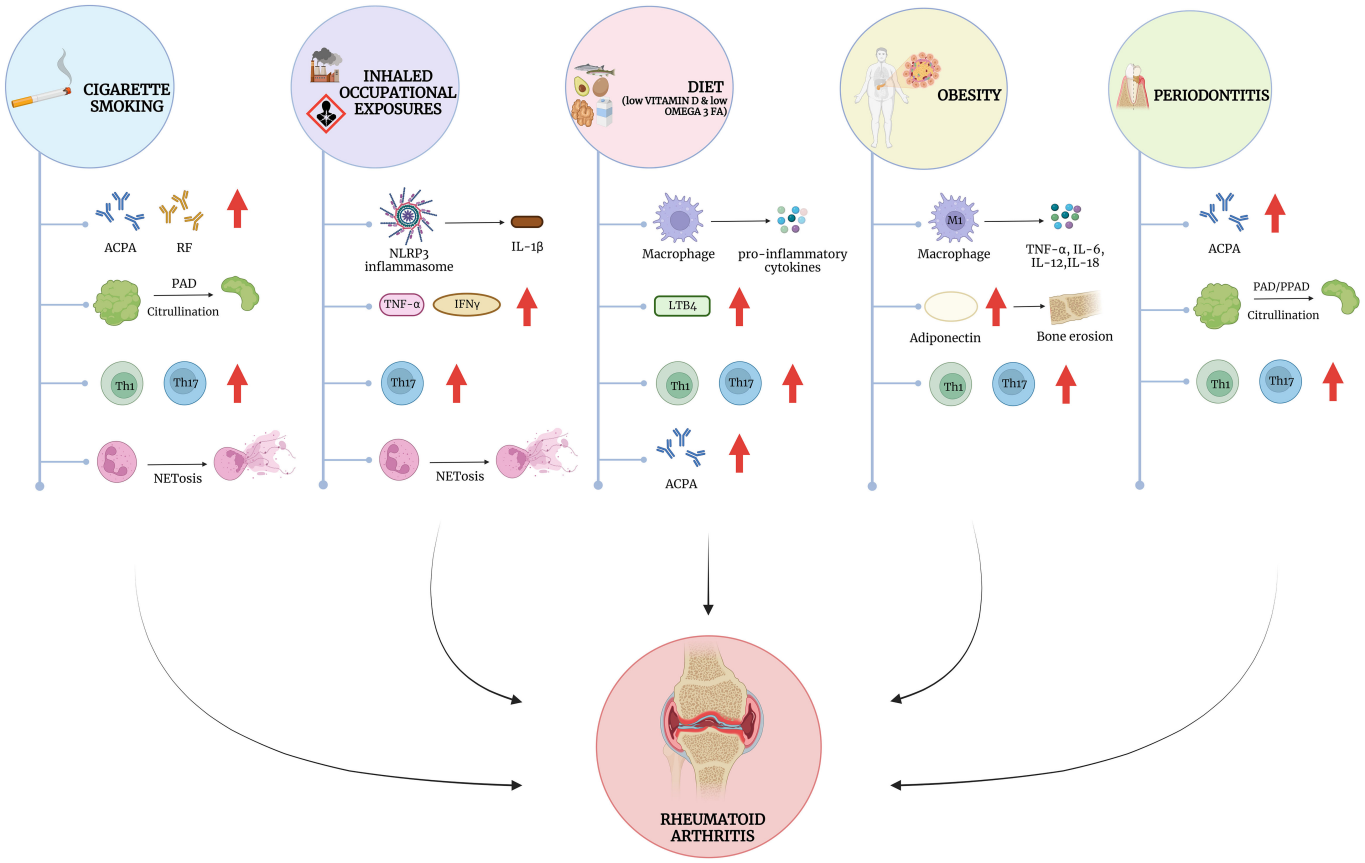
Modifiable risk factors for RA and their impact on autoimmunity and inflammation. Mechanistically, the influence of external risk factors converges on a number of immune cell dysfunction, including the stimulation of the innate and adaptive immune systems. Cigarette smoke has been shown to influence the development of autoantibodies such as anti-citrullinated protein antibodies (ACPA) and Rheumatoid Factor (RF), particularly in the lungs, where it also leads to enhanced citrullination possibly through the activation of neutrophils to form neutrophil extracellular traps (NETosis). Occupational exposures, such as silica dust and textile dust, drives NLRP3 inflammasome activation leading to the release of IL1β, and other cytokines such as TNF-α and IFN-γ. Similar to cigarette smoking, silica may also influence NET formation and the release of citrullinated proteins. Dietary factors such as low vitamin D and Omega-3 fatty acid intake leads to the release of pro-inflammatory cytokines, and eicosanoids such as Leukotriene B4 (LTB4). Obesity has overlapping effects on inflammatory cytokine release with other dietary factors but is also associated with the release of key adipokines such as Adiponectin, which may influence the development of erosive arthritis in RA. Periodontitis shares features with cigarette smoke and occupational exposures, mediating similar processes but in the oral mucosa rather than the lung. Interestingly many exposures strongly influence T-cell polarization, favoring an increase in Th1 and Th17 helper T-cells, which play a crucial role in RA pathogenesis. Created with BioRender.com.

Cigarette smoking is a proinflammatory process, with multiple effects on key immune cells both systemically and at the mucosal surface. Cigarette smoke contains thousands of chemicals including immunomodulators such as nicotine, carbon monoxide, acrolein and oxygen free radicals (58). At the mucosal surfaces, cigarette smoke actives local epithelial cells to produce pro-inflammatory cytokines (59), which enhances immune cell recruitment to illicit an inflammatory response. Here, cigarette smoke may act through pulmonary dendritic cells to enhance T-cell polarization towards Th1 and Th17 CD4 T-cells (60) (**Figure 1**). Furthermore, there is a reduction in regulatory T-cells in the lungs of smokers, although this is not observed systemically (61). *In-vitro* cigarette smoke extract leads to enhanced production of chemokines by monocytes which influences the recruitment of neutrophils (62). This effect is also observed in pulmonary macrophages (alveolar macrophages (63)). Neutrophil chemotaxis is impaired by cigarette smoke extract (64, 65), while their propensity to form NETs appears to be enhanced both *in-vitro* and at the pulmonary surfaces (66). Systemically, cigarette smoking skews the immune system towards a proinflammatory response. Multiple studies have suggested that smoking influences helper T – cell polarization, and in smokers systemic skewing towards Th1 and Th17 CD4 T-cells has been described (67). Interestingly, studies on the immunomodulatory properties of nicotine have pointed to a primarily immunosuppressive effect, predominantly through nicotinic acetylcholine receptors (68). Specifically, nicotine induces T-cell anergy in mice (69) and attenuates collagen induced arthritis (70). Nonetheless, at least one study has pointed to a role for nicotine in worsening murine arthritis, primarily through the formation of NETs (71).

### Inhaled occupational exposures

While cigarette smoke remains the most extensively studied pulmonary exposure associated with RA development, there is also evidence linking RA to other inhaled exposures, particularly those encountered in occupational settings. Case-control studies have shown that silica exposure increases the odds of developing both seronegative and seropositive RA (72–74) (**Table 2**). Furthermore, other inhalants such as pesticides, solvents and other farming related exposures also appear to increase RA risk (88). Air pollution has also been linked to a variety of autoimmune diseases, including RA (78). Textile dust exposure is also linked to RA development, and alike cigarette smoking, displays an important gene-environment interaction with the SE (76). Interestingly, there may be cumulative or synergistic effects of inhalants, with the number of different exposures increasing RA risk in a stepwise fashion. Furthermore, the addition of cigarette smoking and genetic risk to occupational exposures increases the risk of developing seropositive RA dramatically (86, 89). Of the best studied immunopathologic mechanisms, silica dust is not only associated with RA, but there is also clear synergism between silica and cigarette smoke exposure in RA risk (74). Silica is found in nature as quartz, and exposure most often occurs in occupational activities such as mining, drilling and sand blasting. Pathologic exposure to silica occurs primarily though inhalation, where it induces pulmonary fibrosis as a result of inflammation (90–92). Inhaled silica is engulfed by alveolar macrophages, which leads to the production of reactive oxygen species (ROS) (93). Engulfment also leads to rupture of cytosolic lysosomes, which in turn activates the inflammasome and the secretion of IL-1β (94). TNF-α and IFN-γ have been shown to play essential roles in the both the acute and chronic inflammatory responses observed in pulmonary silicosis (95). An exaggerated Th17 response (**Figure 1**), driven primarily by IL-17A production is partially responsible for acute, but not chronic inflammation mediated by silica (96). Pulmonary silica also leads to the recruitment of neutrophils, and the crystals themselves can induce the production of NETs (97). However, silica does not appear to aggravate murine arthritis, and is not associated with an increased production of PAD enzymes in this murine model (98). Regardless, the combination of cellular death, inflammation and activation of lymphocytes may contribute mechanistically to loss of immune tolerance, particularly in genetically susceptible individuals.

**Table 2:** Selected studies showing an association between occupational exposures and Rheumatoid Arthritis (RA) risk.

Exposure	First Author	Study type	Cohort	Outcome	Risk of exposure	# RA cases	Reference
Silica	Wrangel	Case-Control	Sweden	Sero +/- RA	Ever exposed: Sero + RA OR 1.22, Sero - RA 1.23	31139	(72)
Silica	Vihlborg	Retrospective Cohort	Sweden	Sero+ RA	Ever exposed: Sero+ RA OR 2.59	18	(73)
Silica	Ilar	Case-Control	Sweden (EIRA)	Sero+/- RA	Ever exposed: Sero+ RA OR 1.2, Sero - RA OR 1.2	11285	(75)
Textile Dust	Too	Case-Control	Malaysia (MyEIRA)	ACPA+/- RA	Ever exposed: ACPA+ RA OR 2.5, ACPA- RA OR 3.5	910	(76)
Air Pollution	Shin	Case-Control	Korea	RA	O3: RA OR 1.35 to 1.45 (stratified based on quartile exposure)	444	(77)
Air Pollution	Adami	Retrospective Cohort	Italy (DeFRA)	RA	PM10 (high): RA OR 1.4, PM2.5 (high): RA OR 1.6	3817	(78)
Air Pollution	Ho	Retrospective Cohort	Taiwan	RA	PM2.5 (high): RA OR 1.053	9338	(79)
Air Pollution	Zhang	Retrospective Cohort	UK	RA	Combined exposure: RA HR 1.14	2034	(80)
Air Pollution	Shepard	Retrospective Cohort	UK	RA	Any exposure: RA OR 1.15	130	(81)
Coal Mining	Schmajuk	Retrospective Cohort	USA	RA	Any exposure: RA OR 3.5	328	(82)
Coal Mining	Schmajuk	Retrospective Cohort	USA	RA	Any exposure: RA OR 3.6	112	(83)
Rock Mining	Blanc	Retrospective Cohort	USA	RA	Any exposure: RA OR 4.1	89	(84)
Occupational Exposures (multiple)	Ilar	Case-Control	Sweden (EIRA)	ACPA +/- RA	Brick/Concrete: ACPA+ RA OR 2.9, Material handling ACPA+ RA OR 2.4, Electrical ACPA+ RA OR 2.1, Brick/Concrete ACPA- RA OR 2.4, electrical: ACPA- RA OR 2.6	3522	(85)
Occupational Exposures (multiple)	Tang	Case-Control	Sweden (EIRA)	ACPA +/- RA	Any exposure: ACPA+ RA OR 1.25	4033	(86)
Occupational Exposures (multiple)	Noonan	Case-Control	USA	RA	Multiple exposures: RA OR 1.77 to 3.98 (stratified by number of exposures)	129	(87)

Sero+/-, seropositive or seronegative; ACPA, anti-citrullinated protein antibodies; EIRA, Epidemiological Investigation of Rheumatoid Arthritis; RR, Relative Risk; OR, Odds Ratio; HR, Hazards Ratio. All RR/OR/HR are statistically significant unless otherwise stated.

## Dietary Exposures

### Dietary patterns

Over the past few decades, the drastic transformation in dietary habits, particularly the rise of the Western diet, has emerged as a major factor influencing human health and contributing to the escalating burden of chronic diseases worldwide. Diet is considered an important lifestyle risk factor for the development of RA (99). Although an accurate assessment of dietary patterns remains a challenge due to potential confounders and recall bias (100–102), several key studies suggest that dietary factors are implicit in the risk of developing RA (**Table 3**). Studies of diet and RA risk can be split into those analyzing global diet effects (ie. low inflammatory diet, Mediterranean diet, etc.) or the impact specific food groups/nutrients. Unhealthy diets have been linked to multiple autoimmune diseases (112) including RA, multiple sclerosis and inflammatory bowel disease. In RA, unhealthy eating patterns, measured using the Alternative Healthy Eating Index was associated with incident RA cases in the Nurses’ health study (113) even after adjusting for body mass index (BMI). The effects of the Mediterranean diet, a diet that aims to enrich in mono-unsaturated fatty acids, vegetables, fruits and whole grains, is associated with several important cardiovascular benefits (114), has displayed mixed results in protecting against RA development. For example, a large prospective cohort study suggested that adherence to the Mediterranean diet is protective of incident RA in ever smokers (104) after adjusting for BMI. Interestingly, no protective effects were found in non-smokers. In the EIRA cohort, adherence to the Mediterranean diet was also protective for RA, after adjusting for BMI and physical activity (103). Conversely, there are several studies of similar design that have shown no association between the Mediterranean diet and RA risk (103, 115). However, in established RA, randomized controlled trials (RCTs) of Mediterranean diet have shown a reduction in joint disease activity and improvement of function compared to controls diets (116, 117). Diets that are thought to promote inflammation such as a low fibre diet (118), high carbohydrate diet (115), high meat intake (119) have not confirmed any clear association with RA.

**Table 3:** Selected studies showing an association between dietary exposures and Rheumatoid Arthritis (RA) risk.

Exposure	First Author	Study type	Cohort	Outcome	Risk of exposure	# RA cases	Reference
Mediterranean Diet	Johansson	Case-Control	Sweden (EIRA)	RA	Exposure (high): RA OR 0.79, RF+ RA OR 0.69	1721	(103)
Mediterranean Diet	Nguyen	Retrospective Cohort	France (E3N)	RA	Exposure (high): RA OR 0.91 (in smokers)	480	(104)
Fish	Sparks	Retrospective Cohort	USA (Nurses Health Study)	Sero+/- RA	Exposure (high): Sero- RA HR 0.55 (females < 55 yo)	1080	(105)
Fish	Shapiro	Case-Control	USA	RA	Exposure (high): RA OR 0.57	324	(106)
Omega-3 FA/Fish	Di Giuseppe	Retrospective Cohort	Sweden (Swedish Mammography Cohort)	RA	Exposure omega-3 (high): RA RR 0.65, Exposure Fish (high): RR 0.71 (ns)	205	(107)
Vitamin D	Merlino	Retrospective Cohort	USA (Iowa Women’s Health study)	RA	Exposure (high): RA RR 0.67	152	(108)
Vitamin D	Hiraki	Case-Control	USA (Nurses Health Study)	RA	Circulating levels (high): RA OR 0.8 (3 months to 4 years before diagnosis)	166	(109)
Vitamin D	Song	Meta-analysis (3 Retrospective Cohort)	Multiple	RA	Exposure (high): RA RR 0.76	874	(110)
Vitamin D, Omega-3 and combination	Hahn	Randomized controlled trial (secondary analysis)	USA (VITAL Study)	RA	Exposure Vitamin D: RA HR 0.87 (ns), Exposure Omega-3: RA HR 0.80 (ns), Exposure Omega-3 and Vitamin D: RA HR 0.27	45	(111)

Sero+/-, seropositive or seronegative; ACPA, anti-citrullinated protein antibodies; EIRA, Epidemiological Investigation of Rheumatoid Arthritis; RR, Relative Risk; OR, Odds Ratio; HR, Hazards Ratio. NS, not significant. All RR/OR/HR are statistically significant unless otherwise stated.

### Fish and omega-3 fatty acids

While dietary patterns in general have been associated with the development of RA, the role of fish intake, with specific focus on polyunsaturated fats has been a primary focus in RA risk. Fatty fish is rich in long chain omega-3 fatty acids (eicosapentaenoic acid or EPA, and docosahexaenoic acid or DHA) and is the primary dietary source of these fatty acids. Other sources include krill, algae and nuts (120). Fish intake in particular has been linked to protection from RA development (**Table 3**). Outside of the studies that investigated the impact of the Mediterranean diet (104) (linked with increased fish intake), a large registry study from Denmark suggested that high fish oil intake reduces the risk of incident RA (121). Further, high omega-3 and fish intake protected against RA development in a cohort of Swedish women (107). Furthermore, data from the Nurses’ Health Study suggested the impact of cigarette smoking on RA development was attenuated by fish consumption (105). Fish oil supplements have also shown efficacy in prospective trials of patients with established RA, which demonstrates their anti-inflammatory potential (122, 123).

### Fatty acid metabolism and inflammation

Following ingestion, omega-3 fatty acids are stored in the membrane phospholipids and are thought to exert their predominant anti-inflammatory effect following metabolism into eicosanoids. Eicosanoids are a family of molecules, all roughly 20 carbon units in length, called oxylipins which are predominantly made up of several subfamilies including prostaglandins, thromboxanes and leukotrienes. In contrast, omega-6 fatty acids, derived from animal fats, plant oils and cereals are thought to exert pro-inflammatory effects. The ratio of omega-6 to omega-3 is thought to shift the relative contribution of derivative fatty acids towards or away from inflammation. The effect of EPA/DHA on specific inflammatory cells has been studied both *in-vitro* and *in-vivo (* 124). In macrophages, EPA/DHA reduces the secretion of pro-inflammatory cytokines after simulation (125, 126), supresses the inflammasome (127) and increases phagocytic capacity (128). Neutrophils exposed to omega-3 FAs similarly display reduced migration capacity (129), enhanced phagocytosis (130), yet enhanced production of reactive oxygen species (131). Interestingly, RA patients supplemented with omega-3 FA displayed reduced production of neutrophil derived Leukotriene B4, a chemotactic leukotriene (132). Omega-3 metabolites, primarily resolvins, reduce cytokine production in activated CD8+ T-cells, T helper 1 (Th1) cells and Th17 cells, and skews T-cell differentiation away from Th1/Th17 (133) (**Figure 1**), and perhaps towards an increase in Treg cells (134). In asthmatic children omega-3 supplementation reduced IL-17A, a key Th17 cytokine (135). Omega-3 supplementation also reduces the severity of murine arthritis, and this may be in part mediated by effects on T-cell differentiation (134). The effect of omega-3 supplementation on B-cell activation remains less clear. DHA/EPA reduces B-cell activation in human cells treated *ex-vivo* (136). Conversely, in murine models, enhanced IgM production via B-cell proliferation was observed after EPA/DHA treatment (137, 138). Taken together, the impact of omega-3 supplementation appears to impact both innate and adaptive immune responses, and while further study is required, the majority of evidence suggests a prominent anti-inflammatory effect on a wide range of key processes.

### Vitamin D

Due to its immunomodulatory properties, Vitamin D deficiency has been linked to the development of several autoimmune disease, including RA (**Table 3**). In a large cohort study of older women in the USA, Vitamin D supplementation was shown to be associated with a reduced risk of developing RA (108). In a case-control analysis of the Nurses’ Health Study, vitamin-D levels were lower in the months-years prior to RA development (109). A meta-analysis that included over 200,000 participants suggested that high vitamin D intake reduced the risk of developing RA by 24% (110). We have previously shown that Vitamin D levels are significantly lower in ACPA positive first-degree relatives of RA patients (FDR) suggesting a potential role in breaking immune tolerance (139), although similar results were not observed in a USA cohort (140). A vitamin D genetic risk score analysis suggests that specific SNPs related to Vitamin D may be protective for the development of RA-associated antibodies in FDR (141). Several small studies have also suggested efficacy of Vitamin D supplementation in individuals with established RA (142).

### Vitamin D metabolism and influence on the immune system

In order to exert effects physiologically, 1,25-dihydroxyvitamin D (1,25(OH)2D), the active metabolite of Vitamin D, must be synthesized (143). Both Vitamin D2 (ergocalciferol, derived from food/supplements) and Vitamin D3 can undergo conversion by the liver (25(OH)D) and kidneys (1,25(OH)2D). Vitamin D3 is produced in the skin following UVB exposure, typically from sunlight. Vitamin D deficiency is common, particularly in parts of the world where seasonality has a major impact on sun-exposure. Importantly, the immunomodulatory effects of activated Vitamin D(1,25(OH)2D), requires binding to its cognate receptor, Vitamin D Receptor (VDR) which is located in the nucleus where it enacts primarily on the transcription of various genes (144). Vitamin D binding protein (DBP), the major plasma carrier for vitamin D metabolites, plays a crucial role in transporting metabolites to target tissues (145). Importantly, polymorphisms of DBP have been associated with a number of human diseases, including diabetes (146), cancer (147) and RA (146). As such, an important limitation of most of the large studies associating vitamin D status and RA is that they do not take these polymorphisms into account.

Aside from its well-recognized impact on bone homeostasis, vitamin D plays a role in immune homeostasis. In macrophages, activated vitamin D stimulates the production of cathelicidin, an antimicrobial peptide (LL37). Macrophages themselves can hydroxylate 25(OH)D to the active form where local production may allow for a more potent immune response (148). In neutrophils, vitamin D has been shown to enhance bacterial killing, but reduce the production of inflammatory cytokines, thus a dampening of excessive inflammation may be an important effect (149). Upon stimulation, T-cells upregulate VDR allowing vitamin D to supress proliferation and shift T-cell differentiation away from Th17 (150) and Th1 (151) types, favoring the formation of regulatory T-cells which has been shown to influence the development of murine autoimmune diabetes (152) (**Figure 1**). Vitamin D also plays a crucial role in transitioning Th1 cells to an immunosuppressive, IL-10 producing subset (153). Unlike omega-3 FAs, the immunosuppressive effects of vitamin D in B-cells are more apparent. Activated vitamin D reduces the formation of immunoglobulin producing plasma cells following activation (154), along with class-switch memory B-cells by inducing apoptosis (155).

### Obesity

High body mass index (BMI) may also play a role in increasing the risk of RA development. For example, in a case-control study of incident RA, obesity (both BMI and abdominal waist circumference) was associated with an increased risk of RA (156). Several other studies of similar design have also implicated obesity in RA risk (157–159), although studies exist which do not show an association (160) or even a protective role for obesity (161). Interestingly, the risk of obesity and developing RA may be higher in women compared to men (162). Prospective studies of at-risk individuals have also linked obesity with the development of RA, specifically in individuals considered at-risk based on the presence of RA autoantibodies (34), although this association was not found in our study of First Nation FDR (3). Intriguingly, the impact of obesity on RA risk appears to be modified only marginally by physical activity, which is thought to be protective of future RA (163). This points to the complexity of the obesity syndrome, which similar to RA, is an interaction between genetics and environmental factors.

Despite the complexity of obesity, it remains well understood that excessive fat can lead to inflammation through various mechanisms. In obesity, macrophages are skewed towards the pro-inflammatory (M1-like) cells, and away from anti-inflammatory (M2-like) macrophages (150). This plays a key role in perpetuating the inflammatory response. M1-like macrophages display increased secretion of cytokines such as TNF-α, IL-6, IL-12, IL-18, and ROS which are all involved in enhanced inflammatory responses (164–167). These macrophages recruit CD4^+^ T-cells that eventually differentiate into several effector cells, namely Th1, Th2, Th17 and Treg cells (168, 169). Th2 and Treg cells counter obesity-associated inflammation; However, in obesity, these two types of effector cells decrease in number. As a result, the inflammation-promoting cells Th1 and Th17 constitute the majority of the cell population (**Figure 1**). Moreover, adipokines secreted by the adipose tissue are strongly correlated with inflammation (170). Studies have shown that Leptin is increased in early RA (171) and is related to increased ROS production (172). A meta-analysis revealed that Adiponectin is significantly higher in RA patients (9) and may contribute to the bone erosions observed in RA (10) via induction of a pro-inflammatory state in osteoblasts and osteoclasts (11). Other adipokines, such as chemerin, resistin, lipocalin 2 have been associated with clinical outcomes in individuals with established RA (173–177).

## The microbiome

### Gut microbiome

The human microbiome is a vast and intricate ecosystem of microbes residing in our bodies that plays a fundamental role in shaping our health (178). Studies implicate that the gut microbiome have gained ample interest in nearly all fields of medicine. The microbiome has a diverse and still emerging role in digestion and homeostasis of the epithelial layer/mucosal permeability. Further, the influence of diet, medications and other exposures play a crucial role in the overall function and diversity of the microbiota. Dysbiosis is clearly evident in patients with established RA and appears to correlate with disease activity and treatment response (179). Particular interest has been paid to *Prevotella copri (* 180), which is enriched in RA patients and exacerbates murine arthritis (181). Although studies in pre-clinical RA are lacking, a strain of *Subdoligranulum* has been shown to cross react with IgG and IgA from individuals who are at-risk to develop RA (182), although notably, these were not ACPAs. Fascinatingly, when the bacterium is transferred to germ-free mice, it leads to spontaneous development of inflammatory arthritis. The influence of the gut microbiota on inflammatory processes is complex, yet it is clear that the formation of short chain fatty acids (SCFA) from the processing of fiber plays an important role. SCFA are typically thought to be anti-inflammatory, predominantly though the production of regulatory T-cells (183), but also due to altered permeability of the gastrointestinal tract (184). In ACPA+ at-risk individuals with musculoskeletal symptoms, serum SCFAs were higher in those who did not progress, compared to those that developed RA (185). Components of bacteria can trigger toll-like receptors (TLRs), which propagate both innate and adaptive immune responses (186) in resident leukocytes and epithelial cells. Much attention has been paid to segmented filamentous bacteria (SFB), which induces the production and activation of Th17 cells (187) and follicular helper T-cells (188) in the intestine, which influences murine arthritis disease activity (189). Further studies of the role of the microbiome and the risk of RA, with a specific focus on at-risk individuals are required.

### Periodontal disease

Periodontal disease is an oral inflammatory condition by which resident bacteria mediate a cascade of inflammatory responses, ultimately leading to local tissue damage. Periodontitis is a polymicrobial disease whereby overgrowth of oral bacteria interacts with local immune cells leading to inflammation. Similar to RA, inflammatory lesions can lead to radiographic bone loss and irreversible damage (190). To date, the connection between periodontal disease and the development of RA has been shown in a variety of epidemiological studies (191–194). Specifically, a meta-analysis of over 150,000 individuals showed a modest association between RA and periodontitis (195). This association is strongest for ACPA positive RA, and interestingly, RA patients with periodontitis have been shown to have higher concentrations of serum ACPA compared to those without periodontitis (192). Similar to cigarette smoking, there is an association between periodontal disease and the SE that appears to have a strong gene-environment interaction, including leading to more severe/destructive RA (196, 197). Interestingly, treatment of periodontitis in RA patients is associated with improved arthritis disease activity (198, 199). It should be noted however that the co-occurrence of periodontitis and RA does not imply a causative relationship between the two diseases, as there is limited evidence that active periodontitis leads to the development of RA.

### *P. gingivalis* is associated with citrullination and autoantibody responses in RA

The best characterized oral bacteria studied in RA risk is *Porphyromonas gingivalis* (*P. gingivalis*). Of considerable interest, *P. gingivalis* expresses endogenous PAD (PPAD), which can induce citrullination sites of local proteins that are key RA autoantigens (200). Unlike human PAD, PPAD preferentially citrullinates terminal arginine (201) after the digestion of proteins by another enzyme called gingipain (202). Modification of PPAD reduces collagen antibody-induced murine arthritis severity and ACPA levels (203). RA patients have been shown to develop antibodies targeting *P. gingivalis (* 204), and this is observed in the pre-clinical stage of the disease (205). Monoclonal IgG from gingival B-cells cross react with citrullinated peptides derived from human and *P. gingivalis* proteins, suggesting the potential for molecular mimicry (206). Pre-existing periodontitis has been shown to exacerbate the collagen antibody-induced arthritis mouse (207) and the CIA model (208). In the latter, *P. gingivalis* was associated with serum cytokines most suggestive of an enhanced Th17/Th1 response (**Figure 1**). Activation of the innate and adaptive immune responses in periodontitis is crucial. Gingival tissues affected by such inflammation display B and CD4 T-cell infiltration (209), with evidence pointing to Th17 differentiation (210). Neutrophils are recruited to the site of inflammation by IL-8, which expectedly yields the release of destructive, granule proteins, the generation of ROS and the formation of NETs (also yielding enhanced citrullination) (211). A number of other oral microbes, aside from *P. gingivalis*, have been linked to RA development including *Provatella* and *Veillonella (* 212, 213). Recently, evidence of oral microbes traversing into the blood of RA patients which can expose citrullinated antigens to ACPA B – cell and promote inflammation by activating inflammatory monocytes (214).

## Modification of risk factors to prevent or delay RA onset

Although the studies reviewed above link RA development to several important modifiable risk factors, it remains much less clear if modification of these factors meaningfully reduces RA risk, and at what stage of pre-RA this might be most efficacious. Smoking cessation is not only modifiable, but can be aided with medications, cognitive behaviour therapy (CBT) and nicotine replacement. Further, it is associated with other important health benefits such as reduced cardiovascular disease (215) and cancer (216). The only evidence linking smoking cessation and reduced RA risk comes from observational data. For example, in the Nurses’ Health Study, individuals who quit smoking had a 40% reduction in incident seropositive RA. Interestingly, the effects of smoking on RA risk were still detectable 30 years after quitting, compared to life-long nonsmokers (37). A similar finding was observed in a Swedish cohort study, showing a 30% reduction of incident RA following smoking cessation (27). Many clinicians are likely to recommend smoking cessation to RA patients and at-risk individuals, possibly due to the extended health benefits outside of RA risk. However, it remains unclear if smoking cessation following the development of ACPA has any meaningful impact on RA development.

To our knowledge, there are no observational studies linking changes in diet or nutrition and subsequent risk of incident RA. However, in the VITAL study, a trial of Vitamin D and Omega-3 fatty acid supplementation (217) a prespecified subgroup analysis found that the combination of supplements displayed a reduction in incident RA after 5 years of follow-up (111). It’s important to note that because this trial was originally designed for cardiovascular and oncologic outcomes, thus the population was older and likely had average to below average risk of developing RA. Whether Vitamin D or Omega-3 supplementation can be used to prevent incident RA in high-risk groups, such as ACPA positive individuals remains unclear.

RA prevention has recently moved into the forefront of pre-RA research, and a number of prospective clinical trials are complete or ongoing. Most of these trials have deployed re-purposed RA medications, and so far, none have successfully prevented RA, although Rituximab (218), Abatacept (219, 220) and Methotrexate (221) delayed RA onset by several months. It is notable that recruitment to RA prevention trials remains a challenge, and thus choosing the right intervention (222) is crucial to ensure study feasibility which is in line with participants’ views and priorities (223). There are likely many individuals at increased risk of RA who would not accept targeted, potentially toxic immunosuppressive medications to mitigate their risk. There appears to be a willingness to accept lifestyle interventions in ACPA/RF+ individuals with arthralgia (224) and FDR of RA patients (225, 226) to lower the risk of RA, which for individuals with established RA can also help improve disease activity and progression, as outlined by EULAR (European League against Rheumatism) recommendations from 2021 (227). Hence, there remains an unfilled gap in RA prevention care, which is using nutritional, dietary, and lifestyle interventions to evaluate their protective effects in high-risk individuals, either alone or in combination with more targeted drug therapies.

## Further points to consider

When evaluating studies linking modifiable exposures to the risk of RA, it is crucial to consider the strength and levels of evidence supporting the associations. Several limitations apply to retrospective and case-control studies. For instance, even large retrospective population-based studies may lack the power to detect differences due to RA’s relatively low prevalence (~1%). Additionally, how a diagnosis of RA is being made (ACR/EULAR criteria (228), billing codes, self-report) should also be considered and appraised. Case-control studies can be affected by recall bias, specifically with respect to lifestyle exposures. Although prospective cohort studies of at-risk individuals are less common and more difficult to recruit for, they may serve to enrich in the outcome of interest (incident RA), where the prevalence is much higher. However, these cohorts are often smaller, and thus may also suffer from reduced power to detect an association between an exposure and the outcome. Irrespective of the study design, unmeasured or measured confounding factors should be strongly considered as they can influence the interpretation of lifestyle and modifiable exposure studies. For example, many lifestyle factors are interrelated, such as diet and exercise, smoking and alcohol use, and are often connected to socioeconomic status, which itself has been associated with an increased risk of RA (229).

The association between several exposures, including periodontitis, cigarette smoking, occupational exposures all appear to be enriched in seropositive RA, with mixed findings for the strength of association with seronegative RA (**Tables 1**, **2**). Regarding dietary associations, most studies have not stratified based on serological status, but one study found the Mediterranean diet to be protective for seropositive RA (103), and another showed that fish exposure was protective for seronegative RA (105) (**Table 3**). One consideration is the recent discovery of other post-translational modifications which act as autoantigens in RA, such as homocitrulline, acetyl, and MDA/MAA, suggest a potential overestimation or misclassification of seropositive and seronegative disease (230) in many of these associative studies. It is also intriguing that many of these exposures converge on a similar pathway, with a dominant role for mucosal immunity specifically in the mouth, lung and gut. This notion is reinforced by the finding that secretory IgA may play an important role in the generation of pathogenic autoimmunity in RA (231). It remains clear that a deeper understanding of mucosal immunology will be crucial for identifying the origins of RA, particularly seropositive disease.

## Concluding remarks

In conclusion, prospective and retrospective cohort studies have provided key insights into the role of modifiable risk factors and RA risk. Cigarette smoking and diet appear to have substantial impact on incident RA and may be the most practical lifestyle interventions to prevent RA in the future. Further studies are required to understand how these exposures modulate the immune system to promote inflammation, reduce immune tolerance and trigger clinical arthritis in at-risk individuals. Ultimately a combination of prospective clinical trials, longitudinal cohort studies and translational science will help disentangle the key events that drive the development of RA in those at-risk.

## Author contributions

JAM: Conceptualized and wrote the manuscript. Performed literature review. HE-G: Edited manuscript, provided expert advice. LO’N: Edited and conceptualized the manuscript. Performed literature review.
