# Erratum: Modifiable risk factors linked to the development of rheumatoid arthritis: evidence, immunological mechanisms and prevention

**DOI:** 10.3389/fimmu.2024.1370893

**Published:** 2024-02-05

**Authors:** Frontiers Production Office

**Affiliations:** Frontiers Media SA, Lausanne, Switzerland

**Keywords:** rheumatoid arthritis, prevention, environment, risk factors, smoking, diet, mucosal

Due to a production error, the reference list used in the final article was an incorrect earlier version.

The publisher apologizes for this mistake.

The original version of this article has been updated.

